# Refractive Index Sensor Based on a 1D Photonic Crystal in a Microfluidic Channel

**DOI:** 10.3390/s100302348

**Published:** 2010-03-22

**Authors:** Pedro S. Nunes, Niels Asger Mortensen, Jörg P. Kutter, Klaus B. Mogensen

**Affiliations:** 1 Department of Micro and Nanotechnology, Technical University of Denmark, DTU Nanotech, Building 345 East, DK-2800 Kongens Lyngby, Denmark; E-Mails: joerg.kutter@nanotech.dtu.dk (J.P.K.); klaus.mogensen@nanotech.dtu.dk (K.B.M.); 2 Department of Photonics Engineering, Technical University of Denmark, DTU Fotonik, Building 345 West, DK-2800 Kongens Lyngby, Denmark; E-Mail: asger@mailaps.org (N.A.M.)

**Keywords:** 1D photonic crystal, refractive index sensor, integrated waveguides, electrochromatography

## Abstract

A refractive index sensor has been fabricated in silicon oxynitride by standard UV lithography and dry etching processes. The refractive index sensor consists of a 1D photonic crystal (PhC) embedded in a microfluidic channel addressed by fiber-terminated planar waveguides. Experimental demonstrations performed with several ethanol solutions ranging from a purity of 96.00% (n = 1.36356) to 95.04% (n = 1.36377) yielded a sensitivity (Δλ/Δn) of 836 nm/RIU and a limit of detection (LOD) of 6 × 10^−5^ RIU, which is, however, still one order of magnitude higher than the theoretical lower limit of the limit of detection 1.3 × 10^−^^6^ RIU.

## 1. Introduction

In the previous decades, a lot of work has been done on the miniaturization of chemical analysis systems in order to benefit from faster analysis times, reduced reagent consumption and possibly to realize cheaper portable systems. This has resulted in a great increase in the availability of chemical analyses throughout society. However, the detection part of the miniaturized analysis systems remains a considerable challenge, because the traditional methods often scale unfavorably when the dimensions are reduced, due to a reduction in sample volume and optical path length [1,2].

We have developed a waveguide-based refractive index sensor that relies on a 1D photonic resonator for label-free detection in miniaturized separation systems. This sensor differs from the vast majority of waveguide-based evanescent wave sensors, because it utilizes a free-space configuration in order to probe the bulk and not the surface of the solution. Waveguide-based evanescent wave sensors were initially developed for telecommunications and later adapted to biochemical applications [3–5]. However, the majority of chemical analysis systems such as liquid chromatography rely on measuring the bulk of the analyte volume.

An inherent advantage of free-space sensors over evanescent-wave sensors is that all the light that reaches the detector has passed through the liquid [6], enabling higher sensitivities. The higher sensitivities achieved are a direct consequence of a higher overlap between the optical field and the sample, compared to evanescent wave-based sensors [2]. A disadvantage of free-space sensors is the unguided behavior of light in the detection region, which increases the coupling loss over the detection site, compared to evanescent-wave sensors. This results in a higher relative detection noise, which makes it difficult to benefit fully from the higher sensitivity, resulting in a reduced signal-to-noise ratio (S/N). The important parameter from an application point of view is not the sensitivity, but the S/N, so whether an evanescent wave or a free-space sensor is preferred depends on the type of analysis system.

**Figure 1.**
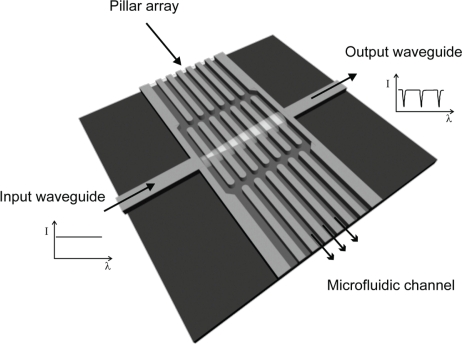
Schematic view of an on-column 1D photonic crystal refractive index chemical sensor. The detection region consists of a pillar array and integrated waveguides coupling infrared light through the resonator.

In our case, the sensor is designed for on-chip electrochromatography systems with microfabricated separation columns [7–10]. The pillar array that constitutes such a separation column is also used as a resonator for on-column label-free detection, where integrated waveguides couple infrared light into/out of the detection site, Figure 1. The advantage of this approach lies in the fact that the detection system imparts no changes to the fluidics, thereby reducing the distortion of the analyte bands in the chemical separation process, and hence achieving a high resolution in the chemical analysis.

The periodicity introduced by the 1D pillar array, in terms of refractive index, symmetry and geometry, gives rise to a band where photons of a particular wavelength range are forbidden to propagate, similarly to the case of electrons in atomic crystals. Typically, this forbidden band is called a photonic band gap and is the reason for the appearance of stop bands in the reflection or transmission spectra, Figure 1.

Photonic crystal sensors are typically fabricated by etching an array of submicron holes in a high refractive index material (e.g., silicon thin film) [11–14]. This is not a viable approach in our case, for two reasons. First of all, using a hole array, transport of the fluids in the plane of the photonic crystal is not possible, hence the sensor cannot be integrated with planar microfluidics with channel dimension in the same range as the microoptical structures. Therefore, a pillar array is used instead. Secondly, silicon with layer thicknesses of several 100 nm is too electrically conductive, thereby not supporting electroosmotic pumping in the final devices due to bubble formation from electrolysis of the buffer solution. However, using a glass instead of a silicon resonator has the disadvantage that the refractive index contrast between the sensor and the analyte is much lower. This issue is addressed by depositing a thin layer of amorphous silicon around the glass pillars to increase the finesse of the resonator. Having such a thin layer prevents bubble formation from electrolysis, because the conductance of the a-Si layer is much lower than the conductance of the buffer solution in the fluidic channel [15].

The initial results on the performance of a similar device have been previously published [6]. In this article, improvements in the optics and fluidics are reported, resulting in two orders of magnitude better detection limit.

## 2. Fabrication

The fabrication of the 1D photonic crystal in a system with integrated waveguides was adapted from our previous work [6]. However, several process modifications were introduced in order to improve the shortcomings of the initial devices. One of the main shortcomings was fluidic leakage along the waveguides. Even though this fact should not influence the optical behavior of the waveguides, it seriously compromised the fluidic handling. Another shortcoming consisted in the non-uniformity of the pillar etching, which influences the spectral shape of the measured resonances.

Optimization of the fabrication process occurred mainly through the waveguide and fluidic channel etching steps, and by improving the sealing of the fluidic channel using a PDMS lid. Etching of the waveguides and fluidic channel was performed in an inductively coupled plasma deep reactive ion etcher (Advanced Oxide Etcher, Surface Technology Systems, UK). Such an etching system enabled the use of resist as a masking layer instead of an a-Si mask for both lithographic steps, thereby simplifying the process.

### 2.1. Waveguide Fabrication

Single side polished 4 inch silicon wafers were oxidized at 1,075 °C for 21 days, leading to a 9 μm thick silicon dioxide (SiO_2_) waveguide buffer layer. Deposition of a 3.0 μm thick silicon oxynitride (SiON) layer followed by a 400 nm thick SiO_2_ layer was done in a plasma enhanced chemical vapor deposition system. The wafer was then annealed for 8 hours in a nitrogen atmosphere at 1,100 °C. UV lithography was used to pattern the waveguides and light blocking structures using a 2.2 μm thick positive resist. Following the resist patterning the waveguides were etched in a deep reactive ion etcher (Advanced Oxide Etcher, Surface Technology Systems, UK), such that a 400 nm overetch would be achieved. The resist used as a mask was then removed in an oxygen plasma and the top cladding layer was deposited on top of the core layer. Etching of the waveguides using a resist mask led to very smooth sidewalls and top facet, essential for low loss waveguides.

**Figure 2.**
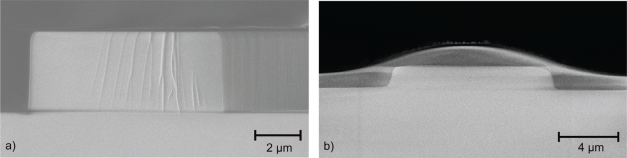
(a) Scanning electron microscope image of 9.0 × 3.0 μm^2^ SiON waveguide after etching. (b) Scanning electron microscope image of 9.0 × 1.5 μm^2^ SiON waveguide after annealing the top BPSG cladding.

Figure 2(a) illustrates the cross section of a 9.0 × 3.0 μm^2^ SiON waveguide after etching and stripping the photoresist mask. The structures on the front facet are due to the cleaving method. Boron phosphorus glass (BPSG) was used as a cladding layer (3.8 μm thickness) as in our initial devices [6]. However, its flowing behavior upon annealing makes it difficult to obtain a perfect seal with a glass lid, due to the uneven surface topography, Figure 2(b). Hence, a different bonding method had to be developed. Annealing of the cladding layer was done in a nitrogen atmosphere for 8 hours at 1,000 °C.

### 2.2. Microfluidic Channel Fabrication

**Figure 3.**
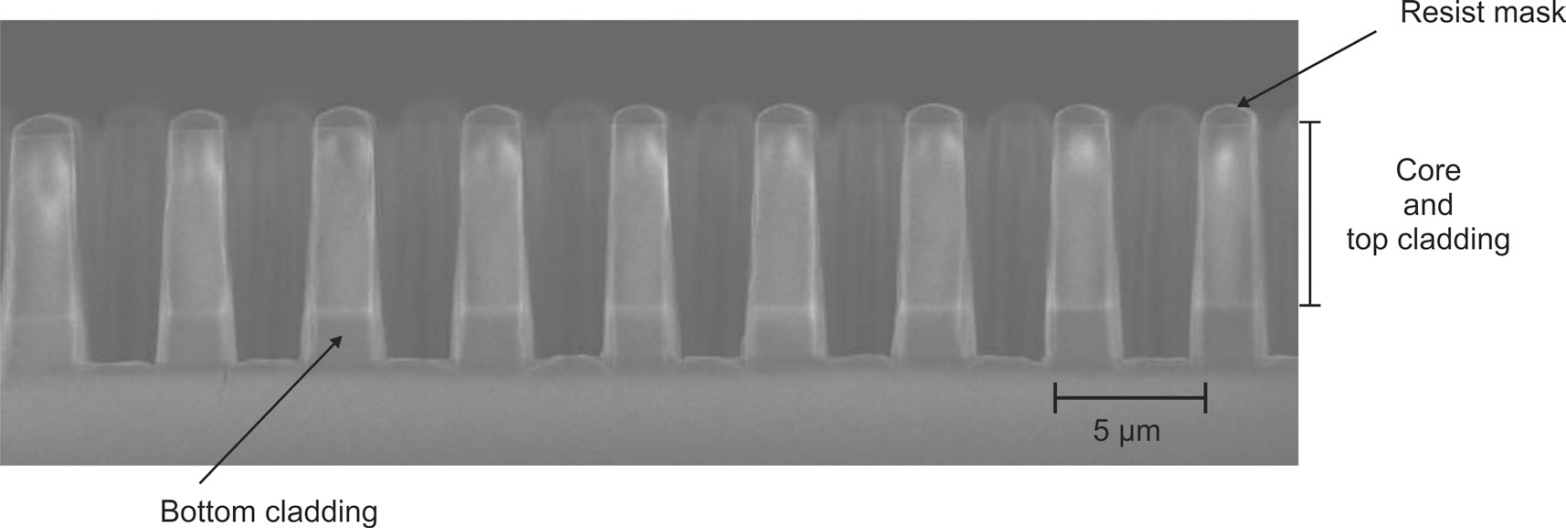
Scanning electron microscope image of the pillar array cross section after etching the core/top cladding and partially etching the bottom cladding, leading to a pillar height of 7.6 μm. The resist mask is still present on top of the pillars, but is partly etched away.

The second lithographic step was performed after the anneal step and similar conditions were used as in the first lithographic step, albeit with a 3.3 μm thick layer of resist. A thicker resist layer was necessary since an 8 μm deep etch was required for the microfluidic/pillar regions in order to go through the waveguide core layer. Figure 3 illustrates a part of the pillar array (before removing the resist mask), where a clear interface between the bottom cladding and the core/top cladding is seen. The pillar array had a periodicity of 5.0 μm and a height of 7.6 μm.

Even though the top surface of these nine pillars is flat, a considerable uneven topography is present close to the walls of the microfluidic channels, which is a consequence of the BPSG cladding’s flowing behavior.

After etching, the resist is once more removed in an oxygen plasma. To improve the resonances a thin layer of a-Si (∼100 nm) is deposited by low pressure chemical vapor deposition. A thin layer was used so that electrokinetic pumping of the fluid is possible without bubble formation due to electrolysis, as mentioned in the introduction.

### 2.3. Bonding

Bonding to a borosilicate glass top wafer proved inefficient to prevent fluidic leakages into the adjacent waveguides regions, thus making it more difficult to pump the fluid in the microchannel. This issue was addressed by bonding a polydimethylsiloxane (PDMS) lid on top of the structured wafer. PDMS is able to fully seal the fluidic channel and prevent leaks along the waveguides, due to its elastomeric properties. In this way, planarization by chemical mechanical polishing was avoided, which would significantly complicate the fabrication process.

The bonding process of a PDMS lid consisted in first removing the air bubbles from a freshly made mixture of PDMS. Then, the air bubble free PDMS was casted on a micromilled PMMA mold with 2 mm wide cylindrical pins for fabricating the inlet and outlet holes. PDMS curing was performed at 80 °C for 1 hour. Before bonding, both the PDMS and the structured silicon wafer surfaces were thoroughly cleaned using isopropanol alcohol and then blow dried with N_2_. PDMS, being an extremely hydrophobic material, required to be plasma activated to increase its surface energy, thus reducing its hydrophobicity. The PDMS and the silicon chip were placed on a chamber and oxidized using 100 sccm of O_2_ for 1 minute at 100 W. After plasma activation, the substrate and the lid were manually aligned and pressed together using a small force for 15 minute and left overnight before injecting any liquid. PDMS recovers permanently its hydrophobicity in a matter of minutes, thus capillary forces are inefficient to pump aqueous solutions through the microfluidic channel and resonator. Hence, filling the microfluidic channel was first done with a concentrated ethanol solution by pure capillary force. Injection of other more aqueous solutions of ethanol was aided by suction.

## 3. Results and Discussion

The devices were characterized by filling the fluidic channel with different ethanol/water solutions (Ethanol 96.00%, n = 1.36356; Ethanol 95.76%, n = 1.36362; Ethanol 95.52%, n = 1.36367; Ethanol 95.04%, n = 1.36377 and Ethanol 94.08%, n = 1.36397), thereby determining the performance of a resonator with eighteen pillars, in terms of its limit of detection and sensitivity. Spectral shifts measurements were preferred to intensity measurements, due to the associated higher noise of the latter measurement method [16]. Highly concentrated ethanol solutions were used solely because of their lower surface tension when compared to more diluted ethanol solutions. A lower surface tension eased the fluidic handling. A 96.00% ethanol stock solution was initially used to characterize the resonator’s optical properties. Infrared light (λ = 1.515–1.544 μm) was coupled in and out of 9.0 μm wide and 3.0 μm high waveguides (five light guiding modes) using single-mode optical fibers (SMF-28e, Corning, USA). The light source consisted of a tunable laser (ANDO AQ4321A) working in a synchronized manner with an optical spectrometer (ANDO AQ6317B).

**Figure 4.**
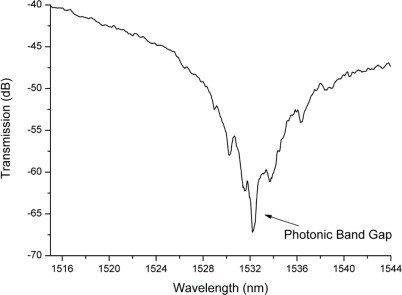
Transmission spectrum of a reference ethanol solution (96.00%) pumped into a microfluidic channel with an 18 pillar array. The 27 dB loss observed at 1532.312 nm corresponds to a convoluted loss of the several modes propagating in the waveguide and not to a specific mode band gap of the resonator.

To achieve a highly stable system and to ease the fluidic handling, single-mode optical fibers were permanently glued (UV glue, Norland Optical Adhesive, USA) to the end facets of the waveguides. Gluing the optical fibers to the multimode waveguides avoids the need for active alignment during the microfluidic handling and ensures that the same optical modes are used for propagation of light in the waveguides. It is necessary to have a high coupling efficiency between the optical fibers and the integrated waveguides to enable the detection of the infrared light transmitted through the 1D resonator. The refractive index of the glue is matched to the refractive index of the fiber core to minimize the coupling losses. A higher coupling loss results in a lower S/N, due to an increase in the noise of the system. High transmission efficiency and high Q-factor are important parameters to make the detection feasible and easy, hence low loss SiON waveguides were integrated on the chip for coupling of the light. Characterization of the waveguides yielded a total insertion loss of 8 dB in the scanning range. The loss in the resonator due to the periodic modulation of the refractive index depended on the wavelength, as can be seen from Figure 4.

The loss caused by the absence of light guidance in the resonator and scattering cannot be measured in a straightforward manner, since it is superimposed on the wavelength modulated loss.

**Figure 5.**
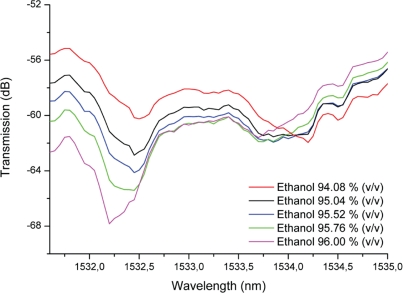
Transmission spectra of the 1D photonic crystal microfluidic channel (eighteen pillars) for five different ethanol concentrations [96.00%, 95.76%, 95.52%, 95.04% and 94.08% (v/v)].

Several experiments were performed by continuously pumping 96.00% ethanol stock solution through the microfluidic channel and measuring the transmission spectra. This enabled the measurement of a considerable dB loss at 1532.312 nm, as shown in Figure 4. The peak measured at 1532.312 nm corresponds to a convolution of the peaks observed for each mode propagating in the waveguide and resonator, but will here, for the sake of simplicity, be addressed as the band gap. A Q-factor of 2,190 was obtained for the center peak present in the band gap. Moreover, emptying the microfluidic channel and filling it again several times with the same solution resulted in a standard deviation of 0.002% (1532.312 ± 0.029 nm). Pumping of fluids with higher refractive indices caused the resonance peaks in the spectra to shift to longer wavelengths, as depicted in Figure 5.

**Figure 6.**
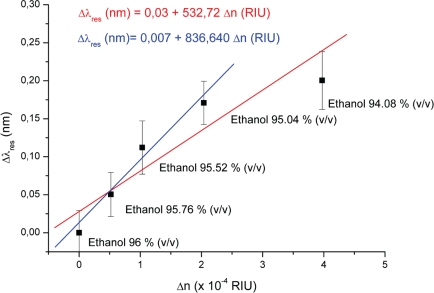
Shift of the resonant wavelength plotted as a function of refractive index shift (Δ*n*). Linear fit of the experimental data yields Δ*λ*/Δ*n* ≈ 532 nm/RIU (sensitivity) for a dynamic range of 4 × 10^−4^ RIU (R^2^ = 0.84). For a dynamic range of 2.1 × 10^−^^4^ RIU, the linear fit of the experimental data yields a sensitivity of Δ*λ*/Δ*n* ≈ 836 nm/RIU (R^2^ = 0.97). The error bars refer to fluctuations on the resonant wavelength after successive injections with the same fluid.

This shift (Δ λ) can be approximated by a linear fit for a determined refractive index range (Figure 6) [17]. The error bars are based on three spectral scans for each solution, while continuously pumping the liquid through the channel. Three reference measurements were performed after each data point in the graph had been measured, by pumping the stock solution through the channel. This reference check was done to ensure that the signal would go back to the baseline, so it could be concluded that the detected refractive index change was not due to a drift in the system. The whole measurement series was performed without re-alignment of the optical system.

The slope from Figure 6 yields a sensitivity of 532 nm/RIU (measured at λ = 1,532.3 nm) for a refractive index interval of 4 × 10^−4^ RIU. The shifts depicted in Figure 6 are approximately on the level of the limit of detection considered (LOD = 2σ), which corresponds to a minimum refractive index difference of 6 × 10^−5^ RIU (0.06 nm shift). The exception occurs for the 94.08% solution of ethanol, where the actual shift is smaller than the considered LOD, which results in a rather low fit quality (R^2^ = 0.84). This deviation can be explained by the multimode behavior of the waveguides and resonator. Performing measurements with multimode waveguides and resonators gives rise to a spectrum that does not correspond to one single propagating mode, but to a convolution of all the spectra corresponding to each effective index (mode). This convolution is not linear in nature, and depends on many factors, for example, the angle of incidence between the mode and the pillar [16,17]. We believe that beyond a refractive index interval of 2.1 × 10^−4^ RIU, as is the case for the 94.08% ethanol solution, the initial propagating modes switch to other propagating modes, as in the analogous case of mode hopping in lasers. Since different propagating modes are used, the data obtained for the 94.08% ethanol solution cannot be considered for the sensitivity evaluation of our device. Thus, we report on a sensitivity of 836 nm/RIU for a refractive index interval of 2.1 × 10^−4^ RIU, which results in a much better fit to the considered data points (R^2^ = 0.97). So far, the smallest refractive index change measured was Δ*n* = 5 × 10^−5^ RIU, for a detection cell volume of 4 pL. One of the main limitations of having several propagating modes in the resonator is the rather limited dynamic range. However, in this way packaging with external optics is easier, due to the increased tolerances when gluing optical fibers to the end facet of the waveguides, hence avoiding the need for active optics alignment.

The limit of detection and the sensitivity are the most crucial parameters for the sensor performance. The sensitivity achieved for a sensor can be very high, but if, for instance, the noise associated with the fluidic handling dominates the system, then no improvement in the LOD of the sensor is assured. The limit of detection is defined as the refractive index for which the smallest resonance shift can be measured in the presence of noise. The experimental noise of the fabricated device has several contributions: fluidic handling (e.g., exchange of the liquid sample and attachment/de-attachment of the pump without any alignment of the optics), thermal fluctuations (since no active control of the temperature was embedded in the chip), detector noise, and fluctuations of the laser intensity. Based on the listed experimental noise sources a more realistic evaluation, resembling a practical application, of the chip LOD can be done. Conversely, several devices in the literature are evaluated based on a calculated LOD, only taking into account the noise of the tunable laser source and the sensitivity of the chip LOD *= λ_resolution_*/(Δ *λ/*Δ*n*) [3,18]. Such a calculation would, for our device, yield a LOD < 1.3 × 10^−6^ RIU, which can be considered as the theoretical lower limit of the LOD and not the real one. In our refractive index sensor, the main parameters available for improving the LOD are the temperature control and the fluidic handling, since the others are intrinsic to the setup. Thermal fluctuations can be addressed by attaching a Peltier element to actively control the temperature of the chip [18] or by devising a sensor which monitors the resonances of a reference branch, thus enabling the subtraction of the thermal fluctuations, such as in a Mach-Zehnder interferometer. Fluidic handling noise could also be minimized by having more efficient connections for delivering and exchanging fluids, as for example demonstrated in the work by Zlatanovic *et al.* [13].

The high sensitivity of 836 nm/RIU depicted in Figure 6 is due to the fact that all light is coupled into the liquid, resulting in a much bigger overlap between the optical field and the liquid compared to evanescent-based sensors. The filling factor (*f*) accounts for the light-liquid overlap in such a system and can, according to Mortensen *et al.*, be expressed as Δ*λ*/Δ*n* = *fλ/n* [2]. According to the calculated sensitivity (836 nm/RIU), a filling factor on the order of 72% has been obtained, which is rather high when compared with [12,18]. An exception to the typically low filing factors achieved for evanescent wave-based sensors is the work developed by Sumetsky *et al.*, where a sensitivity of 800 nm/RIU was achieved [3]. Despite the low dynamic range of our device when compared to [3] the theoretical limit of detection is still on the same order of magnitude (10^−6^ RIU). Wang *et al.* fabricated a sensing microcavity in between two photonic crystal waveguides [17]. Here, a sensitivity of 330 nm/RIU was achieved for a rather large index contrast, but only a minimum refractive index difference of 10^−3^ was measured. Most state of the art commercial refractometers have a resolution up to 10^−^^9^ [19]. Despite the existence of a few sensors with a better performance (lower LOD), the possibilities that the device presented here offers for performing real-time measurements in the bulk solution of a microfabricated chromatography column makes it unique. Moreover, large sample volumes of more than 5 μL are usually required in commercial refractometers [19].

## 4. Conclusions

In conclusion, a refractive index sensor has been integrated in a microfluidic channel to probe the bulk of the analyte. The refractive index sensor consists of a 1D photonic crystal addressed by integrated SiON waveguides. The device differs significantly from many PhC-based sensors because the sensing area is based on pillars instead of holes, which allows liquid transport along the plane of the chip. Moreover, such a device is not limited to surface sensing as it is often the case with photonic crystal sensors. Light is transmitted through the pillar array contributing to a high light-liquid interaction, thus to a high sensitivity (836 nm/RIU). Leak-free sealing was achieved through the use of a PDMS lid, which still supports electroosmotic flow, hence enabling its use as a separation column, which will be addressed in future work.
